# Predicting the expression level of Ki-67 in breast cancer using multi-modal ultrasound parameters

**DOI:** 10.1186/s12880-021-00684-3

**Published:** 2021-10-16

**Authors:** Chen Cheng, Hongyan Zhao, Wei Tian, Chunhong Hu, Haitao Zhao

**Affiliations:** 1 Department of Imaging, First Affiliated Hospital of Soochow University, No. 188, Shizi Street, Canglang District, Suzhou, 215006 China; 2 Department of Ultrasound, Lianyungang Traditional Chinese Medicine Hospital, Lianyungang, 222004 China; 3 Department of Endocrinology, Lianyungang Traditional Chinese Medicine Hospital, Lianyungang, 222004 China; 4 Department of General Surgery, Lianyungang Traditional Chinese Medicine Hospital, Lianyungang, 222004 China

**Keywords:** Breast cancer, Ultrasound, Ki-67, Color Doppler flow imaging (CDFI)

## Abstract

**Objective:**

This study investigated the feasibility of predicting the expression levels of Ki-67 in breast cancer using ultrasonographic findings and clinical features.

**Methods:**

Fifty-eight breast cancer patients, with 82 lesions confirmed by surgical pathology, were selected retrospectively for this study. Conventional ultrasound examination and elastography examination were performed before surgery. Clinical features (age, estrogen receptor (ER), progesterone receptor, and human epidermal growth factor receptor-2 expression levels), ultrasonographic findings, and elastography scores, including the maximum size, location, number, margin, borderline, blood flow, and elastography score of the mass, were collected. The expression of Ki-67 was recorded using immunohistochemical staining, and the patients were divided into a high (≥ 20%) expression group and a low (< 20%) expression group. SPSS 23.0 software was used for statistical analysis. An independent sample t-test was used for measurement data, and a χ^2^ test was used for enumeration data. Logistic regression analysis was performed for meaningful indicators, and the receiver operating characteristic curve was used to calculate the best diagnostic cut-off point.

**Results:**

Monofactorial analysis showed that there was a statistically significant difference (p < 0.05) between the high expression of Ki-67 and the maximum diameter of the mass, the margin of the mass, the color Doppler flow imaging of the blood flow, and the resistance index of the blood flow. There were no significant differences in age, mass location, number, morphology, borderline, microcalcification, and elastography score (p > 0.05). Multiple factor regression analysis showed that a large mass and a mass with a rich blood flow had an independent predictive value for Ki-67. When the diameter was > 21.5 mm, the diagnostic sensitivity and specificity were 91.9% and 71.3%, respectively. The expression level of Ki-67 was negatively correlated with that of ER.

**Conclusion:**

The tumor size and blood flow of breast cancer is correlated with the expression level of Ki-67 and, thus, it could be used to predict the expression level of Ki-67 in ultrasound diagnosis. The margin condition and the expression level of ER of an ultrasonic mass could also indirectly reflect the Ki-67 expression level of the mass.

## Introduction

At present, breast cancer is the most frequently diagnosed cancer in women worldwide, with a yearly increase in its incidence and a high fatality rate. Some studies have shown that breast cancer accounts for 25% of all cancer categories and 15% of deaths related to cancer [1]. With the rapid development of personalized and precision medicine technologies, tumor biomarkers are playing an increasingly important role in clinical management. As a proliferation marker, the Ki-67 index is used clinically to determine the prognosis of cancer and evaluate its treatment, and it is of great importance to decision-making concerning the adjuvant treatment of early breast cancer [2]. There are many breast cancer screening imaging technologies, but ultrasound technology has the advantages of economy, convenience, and strong repeatability. In this study, the intrinsic correlation between ultrasonographic findings and the clinical features of breast cancer and the expression levels of Ki-67 was analyzed to explore the feasibility of using ultrasonic-related indicators in predicting the expression of Ki-67.

## Materials and methods

### Data collection

Patients who received breast cancer surgery in our hospital between May 2017 and August 2020 were retrospectively selected for the study. All the patients received preoperative breast ultrasonography and were pathologically confirmed to have invasive breast cancer after surgical resection of the lesions (58 cases with 82 lesions in total). The patients were 25–71 years old, with an average age of (44.8 ± 11.1) years, and 10 of them had unilateral multiple lesions (≥ 2), 4 had bilateral multiple lesions (≥ 2 tumors on both sides), 3 had bilateral single lesions, and the remaining 41 patients had unilateral single lesions. No radiotherapy, chemotherapy or puncture examinations were performed before the surgery. I confirm that I have read the Editorial Policy pages. This study was conducted with approval from the Ethics Committee of First Affiliated Hospital of Soochow University. This study was conducted in accordance with the declaration of Helsinki. Written informed consent was obtained from all participants.

### Ultrasonography

The LOGIQ E9 Color Doppler Ultrasound Diagnostic System (General Electric Company), equipped with elastic quantitative analysis software and a ML6-15 linear array probe, was used in this study. Patients were examined in the supine and lateral decubitus positions, and the four quadrilaterals of the breast and the axilla were routinely examined. The focus was placed on the evaluation of the size, internal and rear echo, borderline, margin and shape of the mass, the color Doppler flow imaging (CDFI) blood flow grade, and the elastography score. The ultrasound and the diagnosis were performed by two qualified sonographers with at least 5 years of work experience.

### Immunohistochemistry

All the mass specimens were paraffin-embedded and then cut into white slices. The expressions of the breast cancer biomarkers human epidermal growth factor receptor-2 (HER-2), estrogen receptor (ER), progesterone receptor (PR), and Ki-67 were detected using the immunohistochemistry two-step method. With respect to ER and PR, those that were ≥ 10% were determined as the positive expression group, and the rest were the negative expression group, while with respect to HER-2, both (−) and (+ +) were determined as the negative expression group, and (+ +  + +) was determined as the positive expression group. Ki-67 positive was defined as follows: ten cells with light yellow to brown-yellow nuclei were randomly selected and counted at 400-fold microscopes, and the percentage of Ki-67 positive cells in the total cells was calculated. In this study, Ki-67 ≥ 20% was determined as a Ki-67 positive expression, and < 20% was determined as a low-level expression.

### Statistical analysis

SPSS 23.0 software was used for the statistical analysis. For the univariate analysis, the measurement data with a normal distribution were represented by $\overline{\chi }$ ± s, while an independent sample t-test was used for comparison between two groups. The enumeration data was expressed by frequency, and the comparison between two groups was tested with a $\chi^{2}$ test. Statistically significant variables were screened out on the basis of univariate analysis, and then multivariate logistic regression analysis was used to calculate the odds ratio (OR) and the 95% confidence interval (CI) with $\alpha$ = 0.05 as the test level in the forward mode. A p-value < 0.05 was seen as a statistically significant difference.

## Results

### The comparison of the ultrasonographic findings and the clinical features of the Ki-67 expression groups

Univariate analysis showed that there was no statistically significant difference in age, mass location, number, morphology, borderline, microcalcification, and elastography scores between the Ki-67 high expression group and the Ki-67 low expression group. However, the differences in the maximum diameter, margin, CDFI, and resistance index (RI) between the high expression group and the low expression group did have a statistical significance (p < 0.05) (see Table 1).

**Table 1:** Comparison of the characteristics of ultrasound image and clinical features of Ki-67 high expression group and low expression group ($\overline{x}$ ± s)

Variable	High expression group (n = 43)	Low expression group (n = 39)	$^{{\chi^{2} }}$(*t*)	P-values
Age (years)	51.2 ± 9.7	47.6 ± 11.1	1.59	0.12
Position F				
Outer upper quadrant	10	11		
Outer lower quadrant	7	7		
Inner upper quadrant	12	5	3.28	0.51
Inner lower quadrant	5	4		
Nipple dizzy rear	9	12		
Tumor size (mm)	18.7 ± 9.1	12.3 ± 8.6	3.29	0.001
Number of lumps				
1 lump	21	20		
2 lumps	3	7	1.50	0.47
≥ 3 lumps	3	4		
Shape				
Regular	13	14		
Irregular	30	25	0.297	0.586
Border				
Clear	11	17		
Not clear	32	22	2.949	0.086
Edge				
Smooth	10	18		
Not smooth	33	21	4.769	0.029
Microcalcification				
Positive	19	12		
Negative	24	27	1.566	0.211
CDFI grading				
0–I	15	26		
II–III	28	13	8.264	0.004
RI				
≥ 0.7	24	13		
< 0.7	19	26	4.174	0.041
Elastography scores				
1–3	13	20		
4–5	30	19	3.768	0.052
ER				
Negative	16	35		
Positive	27	4	24.005	0.000
PR				
Negative	20	32		
Positive	23	7	11.134	0.001
Her-2				
− ~ +	25	33		
+ + ~ + + +	18	6	6.925	0.009

### Further analysis of omics characteristic variables

**Fig. 1:**
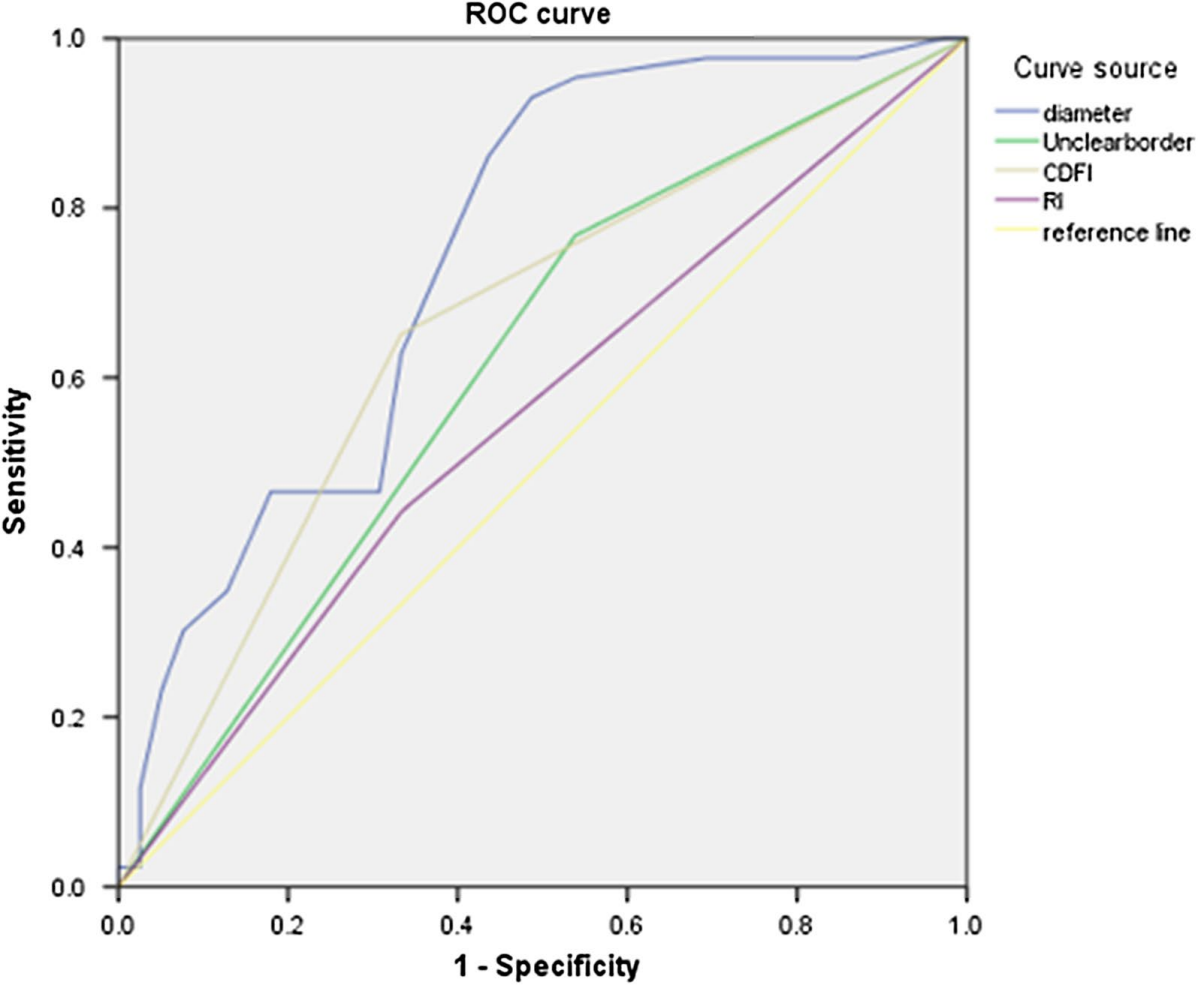
The prediction of the expression level of Ki-67 using the logistic regression analysis of certain ultrasonographic parameters

Use SPSS 23.0 software to perform logistic regression analysis and obtain ROC curve. Multivariate logistic regression analysis was used to further analyze the significant variables, and the analysis showed that the maximum diameter of the mass and the CDFI blood flow had a high prediction value for Ki-67. A receiver operating characteristic (ROC) curve analysis was performed on the mass diameter value, and the area under the curve was 0.744. When the maximum diameter of the mass was > 21.5 mm, the sensitivity and specificity of the high expression of Ki-67 were 91.9% and 71.3%, respectively (see Fig. 1).

### The correlation between the expression of Ki-67, mass diameter, and CDFI

**Fig. 2:**
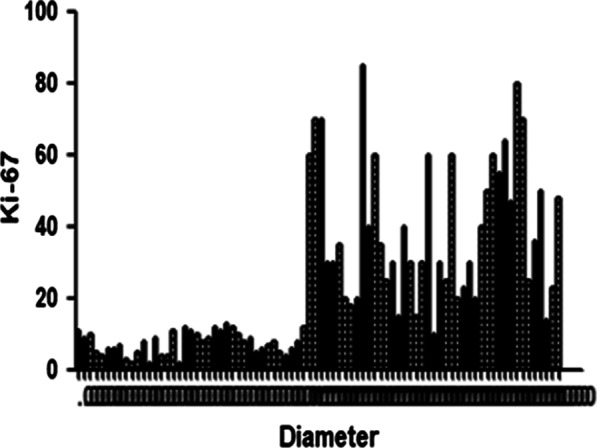
The analysis of the correlation between Ki-67 expression and the mass diameter value

When both the expression level of Ki-67 and the mass diameter were continuous variables, Pearson analysis was used for the correlation statistics of Ki-67 positive values and mass diameter, and the results showed a positive correlation (r = 0.230, p = 0.000) (see Fig. 2). The expression level of Ki-67 was statistically analyzed in the form of continuous variables, and the CDFI score was used as a classification variable, using Spearman correlation analysis, and the results showed a positive correlation (r = 0.276, p = 0.000).

### The correlation between the expression of Ki-67, ER, PR, and HER-2

Univariate analysis showed that the differences between the two groups in the expression levels of Ki-67, ER, PR, and HER-2 were statistically significant (p < 0.05) (see Table 1), while Spearman correlation analysis showed a negative correlation between the expression of ER and that of Ki-67 (r =  − 0.537, p = 0.024).

## Discussion

### The reasons for studying the relationship between the expression levels of Ki-67 and the parameters of ultrasonographic findings

As a biomarker reflecting cell proliferation, Ki-67 has been used to predict the prognosis of cancer and evaluate any therapeutic effect. Recently, there has been a large number of studies on the Ki-67 marker index and its role in cancer, and it has been found that the expression level of Ki-67 increases successively from benign breast tumors, to ductal carcinoma in situ, and then to invasive breast cancer, and this is believed to be related to the occurrence, development, recurrence, and metastasis of some tumors. In normal breast tissues, the expression level of Ki-67 is extremely low (< 3%), and it is not expressed in ER-negative cells [3]. So, a poor prognosis in clinical practice is usually associated with a high expression of Ki-67, but patients with a high expression of Ki-67 may have a better response to chemotherapy. In the past few decades, ultrasonic medicine has seen a comprehensive multi-modal development trend. Compared with other imaging techniques, ultrasound has the advantages of non-invasion, non-radiation, strong repeatability, economy, and convenience, and, thus, it has become the preferred imaging method for breast cancer screening. However, although there have been studies to investigate whether there is a clear correlation between the characteristics of breast cancer ultrasonography and biological prognostic factors, at home and abroad, the research results have not been either conclusive or widely discussed.

### The comparison and analysis of Ki-67 expression, ultrasonographic characteristics, and clinical features

In this study, a large breast cancer lesion diameter (> 21.5 mm) and CDFI blood flow grades were studied as independent predictors. The diameter of the breast cancer mass was used as a morphological index to describe the lesion. It was found that the diameter of a breast cancer mass was positively correlated with the expression level of Ki-67, and ROC curve analysis showed that the high expression of Ki-67 was more likely when the diameter was > 21.5 mm. This conclusion is consistent with previous research [4]. It is thought that Ki-67 plays an important role in the process of cell proliferation and differentiation and has a positive correlation with vascular endothelial growth factor (VEGF). The main function of VEGF is promoting angiogenesis, which increases the permeability of blood vessels and their "nutrition" supply. This causes a mass to grow faster and increase in size with a higher degree of differentiation although its growth mode is prone to longitudinal growth. At the same time, in a state of high expression of tumor immune factor Ki-67, the proliferating cells are accompanied by new blood vessels, with the blood vessel density increasing, resulting in a rich blood flow and a high CDFI grade [5]. However, in current clinical studies, conclusions concerning the correlation between ultrasonography and certain immune factors have not been consistent. Most research suggests that rear echo attenuation is positively correlated with ER, a mass diameter > 2 cm is positively correlated with HER-2 and Ki-67, and a high CDFI score and lymph node metastasis are negatively correlated with ER and positively correlated with HER-2 [6, 7], findings that are consistent with most of the results in this study.

In addition, this study showed that the margin condition of the mass and the RI in the blood flow were also correlated with the difference in Ki-67 expression between the two groups in the univariate analysis, but they were not independent factors to predict the expression level of Ki-67 after multivariate analysis, which may indicate that their predictive ability is not strong or is weakened in comparison. However, related studies [8] have shown that the margin zone of breast cancer is often the area of proliferation and infiltration, and the expressions of Ki-67 and VEGF play a synergistic role in the occurrence and development of tumors. The proliferation of tumor cells requires the nutritional support of neovascularization, so the mass is prone to having an unclear margin, especially in contra-enhanced ultrasound.

In elastography, "hardness" has been introduced as a new parameter in recent years, providing a new clinical reference index. The "hardness" of the mass is mainly manifested as the formation of calcification in two-dimensional ultrasonography. Some imaging studies found that the microcalcification of the breast cancer mass was a negative prognostic factor for Ki-67 [9]. In this study, however, there was no statistically significant difference in the expression level of Ki-67, the elastography score, or the microcalcification signs between the two groups. As a new technique of elastography, shear wave elastography (SWE) is more objective and reliable than the ultrasound elastography scoring method, and it has a good application value for the identification of Breast Imaging-Reporting and Data System 4 category breast lesions [10]. There have been some studies on the correlation between SWE parameters and the expression level of Ki-67 that have found that the size of the elastography ratio was correlated with the expression of Ki-67 [11]. Ye et al. found a statistically significant difference in the elastic modulus of SWE in the transverse, longitudinal, and 45° directions for masses < 2 cm [12]. However, as to whether there is a certain relationship between the multiple parameters of SWE and the molecular typing of breast cancer, the diagnostic criteria have not been agreed at home or abroad, and further studies are required with an expanded sample size.

The correlation analysis of Ki-67, ER, PR, and HER-2 showed that the expression level of ER was negatively correlated with that of Ki-67 (r = − 0.537, p = 0.024). This could have been because the expression of ER inhibited the vascular pathway, leading to the decreased blood perfusion of the mass, in contrast to the rich blood supply of Ki-67. Moreover, tumors with low ER expression have been found to be more aggressive, with a poor prognosis, and they are not sensitive to endocrine therapy [13, 14]. However, some researchers believe that the highly expressed masses of the two are both rich in blood flow and have a positive correlation [15], so further studies are needed, with a larger patient sample, to confirm this.

### Limitations

This study has a number of limitations. First, as breast cancer is a multi-gene disease, more biomarkers may be required for diagnosis assessment and early clinical detection, as well as for evaluating the prognosis and determining a postoperative treatment plan. Thus, further research into biomarkers is required to provide a deeper clinical understanding of the molecular biological behavior of breast cancer. Second, there are various pathological types of breast cancer, but only invasive breast cancer was looked at in this study, so its representation was limited. Thirdly, it is emphasized here that research data may be affected by various uncertainties and/or inaccuracies, and further research will use fuzzy preprocessors on research data [16, 17]. Finally, this was a single-center data study with a small sample size and a limited set of parameters under investigation.

## Conclusion

In conclusion, the size of the breast mass (D > 21.5 mm) and the internal blood flow in breast cancer ultrasonography are correlated with the expression level of Ki-67 to a certain extent, which means the expression level of Ki-67 can be predicted in an ultrasound diagnosis. The margin condition and the expression level of ER of the ultrasonic mass may also indirectly reflect the expression level of Ki-67 to a degree. In future clinical practice, the prognosis of breast cancer can be preliminarily evaluated using ultrasonographic characteristics, thus enhancing the application value of ultrasonography in the clinical diagnosis and treatment of breast cancer [18].

## Data Availability

The datasets used and/or analysed during the current study available from the corresponding author on reasonable request.

## Abbreviations

ER: Estrogen receptor
CDFI: Color Doppler flow imaging
HER-2: Human epidermal growth factor receptor-2
PR: Progesterone receptor
CI: Confidence interval
RI: Resistance index
ROC: Receiver operating characteristic
VEGF: Vascular endothelial growth factor
SWE: Shear wave elastography
